# Enrolling Women into HIV Preventive Vaccine Trials: An Ethical Imperative but a Logistical Challenge

**DOI:** 10.1371/journal.pmed.0030094

**Published:** 2006-02-21

**Authors:** Edward Mills, Stephanie Nixon, Sonal Singh, Sonam Dolma, Anjali Nayyar, Sushma Kapoor

## Abstract

Mills and colleagues argue that the challenge of enrolling women into HIV vaccine trials must be overcome in order for trials to be considered ethical, valid, and generalizable.

With almost 5 million new HIV infections and 3 million deaths from AIDS occurring every year worldwide, the development of a safe, effective, and accessible HIV vaccine has become one of the most urgent global public health needs. The United Nations estimates that 17.5 million women between the ages of 15 and 49 years are living with HIV, accounting for nearly half of the 40.3 million infections worldwide [ 1]. These figures reveal the increasingly female face of AIDS.

As with many diseases, women in developing countries are particularly vulnerable to HIV. Poverty—and women's necessary reliance on men for economic subsistence—may force some women to exchange sex for money or material favors. Women in some cultures lack access to information on protection from and treatment of sexually transmitted infections. There may be societal pressures for women to have children, thereby affecting their use of contraception. Also, in many settings, women and girls lack the ability to negotiate safe sex or demand fidelity in a relationship. For most women in these situations, it is not their own behavior but the behavior of their partners that puts the women at risk of sexually transmitted infections, including HIV [ 2]. The possibility of a preventive HIV vaccine, therefore, holds tremendous promise for women.

Despite the epidemiologic reality, women have had minimal participation in HIV vaccine trials [ 2]. To develop HIV vaccines with regional efficacy, it is important to identify and characterize the viruses that are transmitted, in particular to individuals living in areas and conditions of high incidence. Enrolling women in HIV vaccine trials represents an important challenge that must be fulfilled in order to conduct ethical, valid, and generalizable trials [ 3– 5].

## Women's Concerns about Enrolling in HIV Vaccine Trials

Efforts to enroll and retain women in trials begin by recognizing that their expectations and requirements for participation may be different from those of men. Women may lack the decision-making freedom to participate in a trial, especially a trial that addresses sexual behavior. They may be burdened with childcare and a lack of transportation. For women with children, participation is often limited by having to attend one of the few trial sites that offer childcare [ 6]. Indeed, trials requiring that pregnancy and breast-feeding be avoided may place undue stress upon participants in cultures that place value on women's fertility.

Some cultural barriers identified in the recent HIV vaccine candidate trial in Kenya included women's belief that a woman of childbearing age who uses contraceptives is giving her husband an excuse to look for another woman with whom to bear children [ 7]. On the other hand, men believed that childbearing was a way of keeping women from infidelity. Condoms, which were recommended for use during the trial, were perceived as instruments to promote extramarital relationships.

To identify and tackle such barriers ( Box 1), trial staff require gender-specific guidance and training, while recruitment materials should be geared toward both sexes. Supplying trial centers with counselors and staff who are sensitive to gender, class, and cultural barriers may improve women's access to HIV vaccine trials. Allowing flexible clinic hours to meet the specific needs of patients is one of many pragmatic solutions resulting from such sensitivity training.

## Fear of Adverse Events

Participants in HIV vaccine trials often fear that they will become HIV-positive through participation, either through infection from the vaccine or from antibodies produced by the vaccine, which would test positive in some test kits [ 8–10]. A specific female concern is the unknown effects on future pregnancies [ 11, 12]. This fear is compounded by the concern among women that they may be unable to travel or to attain insurance or employment if they test positive as a result of the vaccine [ 8].

At least some of these fears could be allayed by community participation in the form of community advisory boards and the engagement of local community representatives in designing educational materials to educate the medical community and population at large [ 13].

## Informed Consent

Obtaining consent is a further challenge in some female populations, as women in poorer countries often lack formal education and may not understand the uncertainty that exists within clinical trials (the therapeutic fallacy) [ 14]. The principle of informed consent is that consent is freely given, without coercion from trialists or the local community [ 15]. Therefore, all trial-related information should be presented in the local language, and should address varying levels of education in both written and oral presentation so that participants fully understand their rights, risks, and potential benefits. Participants need to be questioned on their understanding of the trial process [ 2]. The informed-consent sheets should be prepared in consultation with the community advisory board and piloted within the target community to ensure gender and social sensitivity.

## Confidentiality

The International AIDS Vaccine Initiative ( http://www.iavi.org) has elucidated the basic requirements of the physical setting required of trial sites ( Box 2) [ 2]. However, ensuring confidentiality is a challenge in centres that are in public view. For women, any breach of confidentiality can lead to increased discrimination and harassment. Women may be subjected to violence or abandonment by their male partners or to discrimination from their employers if they are seen entering trial centres. Other implications include the refusal of sex as a result of a woman's presumed risky lifestyle [ 16]. Safeguards to maintain confidentiality must therefore be in place. Diagnostic tests should only be disclosed to the participant, and supportive counseling should be provided before and after the tests, regardless of the frequency of HIV tests required.

## Barriers Faced by Sex Workers

Sex workers, due to the nature of their work, experience additional concerns. In addition to difficulties related to informed consent, confidentiality, and fear of infection, sex workers may experience continual exposure to coarse client interactions and violence. Sex workers thus require services that address both domestic abuse and client-related violence. They should also receive educational sessions on common myths about safe sex, including false information that may be given to them by their employers or partners [ 17]. Education efforts regarding safe sex should not just involve the trial participants but also their clientele. To accomplish this, the promotion of condom use and safe sex in the commercial sex districts and bars frequented by the clientele is required. Studies have shown that many women require permission from their partners and employers to undergo HIV testing, at times at the risk of violence [ 18, 19]. Education aimed at sex workers' partners and employers could make this process more socially acceptable.

## Benefits of Participation

Considering the social risks of HIV vaccine trial participation, the immediate benefits to women are small; efforts to recognize their participation begin by appreciating the value of their role. The benefits that might arise from participation include the potential that HIV education may reduce the risk of infection and that participants might receive health care and contraceptive advice that would not normally be provided in communities where health services are limited. We should, however, recognize that despite education on safer sex for women, safe sex is most often determined by their partners' behavior [ 2].

Conducting valid and generalizable HIV vaccine trials requires the equitable inclusion of women. Barriers for participation of women are often systematically different from barriers for men, since the barriers to women stem from their often lower social status and lack of decision-making rights. Due to the number of different HIV clades, trials need to be conducted in a variety of cultures and classes. Although differences in women's rights exist between varying cultures [ 20], most often in settings with high HIV prevalence, women suffer from a lack of empowerment in health and the possibility of violence or social discrimination for being involved with people living with HIV/AIDS or participating in a trial of this unmentionable subject.

In order to conduct clinically meaningful subgroup analyses, a large enough sample of the planned subgroup must be available, with adequate power to detect an effect. To ensure adequate power within the trial participants, the participants must be at risk of infection [ 21]. Thus, enrolling females at high risk in HIV-endemic regions allows room to make important clinical inferences, but puts these individuals in potentially risky situations [ 22].

Recommendations to support gender recognition and sensitivity are often provided for staff allied to the trial (such as coordinating clinic staff). Efforts to create a specific, gender-sensitive, and informed consent process are undermined if the manner with which the consent is presented or obtained is not respectful and inclusive of the target community. Gender advisors and community advisory boards made up of key informants from the risk groups can inform education efforts aimed at potential trial participants [ 2]. We acknowledge that cultural differences in women's rights can be extreme, and that in-depth knowledge of the community, where the trial is being conducted, is imperative to understanding barriers to participation. This article, and those identified elsewhere [2,9,12, 23–28], should be considered basic reading in preparation for designing trial protocols and recruitment strategies.

Recruitment efforts to include at-risk women in HIV vaccine efficacy trials are diverse, and require active involvement of community agencies. Successfully retaining these women over time presents ongoing challenges that relate to the trial validity, which will need to be addressed to ensure women's involvement in future trials [ 29]. The AIDS Community Health Initiative Enroute to a Vaccine Effort (Project ACHIEVE), a vaccine preparedness study in New York City's South Bronx area ( Figure 1), successfully retained 92% of women enrolled after one year [ 24]. Similar retention rates have been replicated in HIV vaccine trials with similar cohorts of women in New York. Concerns about retaining hard-to-reach populations should not cause the exclusion of high-risk women from HIV vaccine and other prevention trials [ 30].

**Figure 1:**
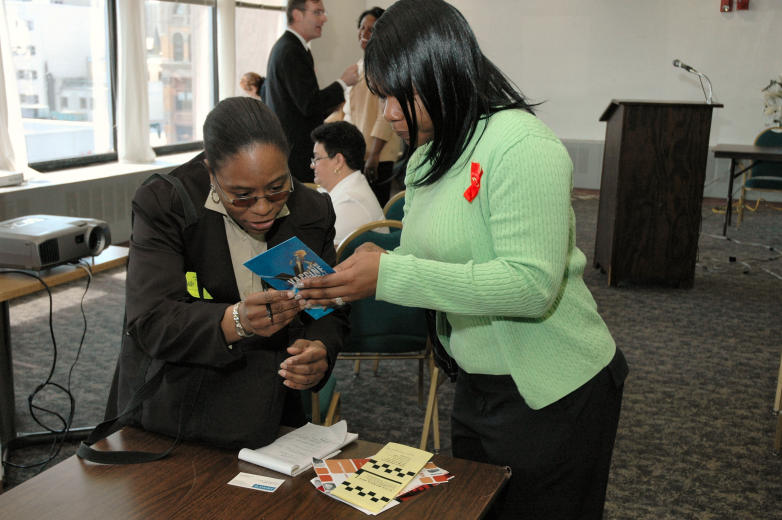
HIV Vaccine Awareness Day at the AIDS Community Health Initiative Enroute to a Vaccine Effort (Project ACHIEVE), 18 May 2005 (Photo: Project ACHIEVE)

The recent cessation of the tenofovir pre-exposure prophylaxis trials in sex workers in Cambodia and Cameroon demonstrates the difficulties of ensuring the rights of the enrolled participants [ 13, 31, 32]. The trials closed early due to widespread complaints that the participant communities had not been involved in the planning of the trials. The events that halted the trials exemplify the need to involve the target groups in the planning of prevention trials [ 33].

## Researchers as Human Rights Advocates

We (EM, SS) recently advocated for the development of standards for community advisory boards [ 13], such as exist for ethical review committees, so that efforts to engage the target populations will transcend tokenism. We recognize that this concept will be new for many clinical trialists. We believe that researchers, by the nature of their work, should be advocates for the rights and protection of trial participants, and in communities and countries where the rights of the participants are threatened, researchers should determine if it is appropriate to engage in research there and seek assistance from human rights monitors when there is uncertainty. The development of trial protocols needs to consider accounts of violence and human rights violations, and develop a strategy for improving conditions for individuals or communities affected.

Today's AIDS research uses technologies that may be exciting from a scientific standpoint but are challenging when we consider the risks participants may incur by participation. Many high-risk communities are ready to assist in research, but researchers must be prepared to assist the communities beyond the trial's duration and ensure that local standards of medical care are improved through the research itself and also through the presence of researchers who advocate on participants' behalf.

## Conclusion

Enrolling women in HIV vaccine trials worldwide represents an important challenge. Ensuring that the rights and needs of this population are respected and met requires community involvement and representation. Researchers must be sensitive to the needs of high-risk and vulnerable groups, from the initial stages of the trial through to unforeseen future events. By implementing strategies to enroll and protect the high-risk and vulnerable participants, we can appreciate the enormous contributions they are making to science.

**Box 1. Important Barriers to Enrolling Women in HIV Vaccine Trials**

Women's enrollment may be hindered by their fears or concerns about the following:

- Contracting HIV from the vaccine
- Testing positive for antibodies to HIV
- The effect of the vaccine upon future pregnancies
- Appearing to distrust one's partner
- Mistakenly being viewed as HIV-infected
- Their partner refusing sex due to the woman's involvement in the trial
- Inadvertent disclosure of trial participation
- Discrimination against participant by family
- Being refused entry into countries, or difficulties with immigration, if they test positive for antibodies to HIV
- Potential job loss
- The possibility of receiving a placebo
- Being able to obtain insurance if they are infected during the trial
- The lack of convenient clinic hours for mothers, houseworkers, and sex workers

**Box 2. Basic Requirements to Make the Physical Environment of the Trial Site Gender-Sensitive**

- Convenient location for women to attend
- A reception area and space that would be unintimidating, welcoming, and volunteer-friendly
- Privacy—in terms of being neither seen nor heard—when interviews are conducted
- A waiting area with general space for families and a specially designated area for women and children
- Childcare during medical examinations or counseling sessions
- Clean toilets, a canteen, and appropriate audiovisual material and educational literature

(Reproduced, with permission, from [ 2])
